# Supplementary material for: Long-Term SARS-CoV-2-Specific Immune and Inflammatory Responses Across a Clinically Diverse Cohort of Individuals Recovering from COVID-19
Source: medRxiv. 2021 Mar 1:2021.02.26.21252308. Preprint. [Version 1] doi: 10.1101/2021.02.26.21252308 (PMC7941662; doi:10.1101/2021.02.26.21252308)
Supplement: Supplement 1 [file media-1.pdf]

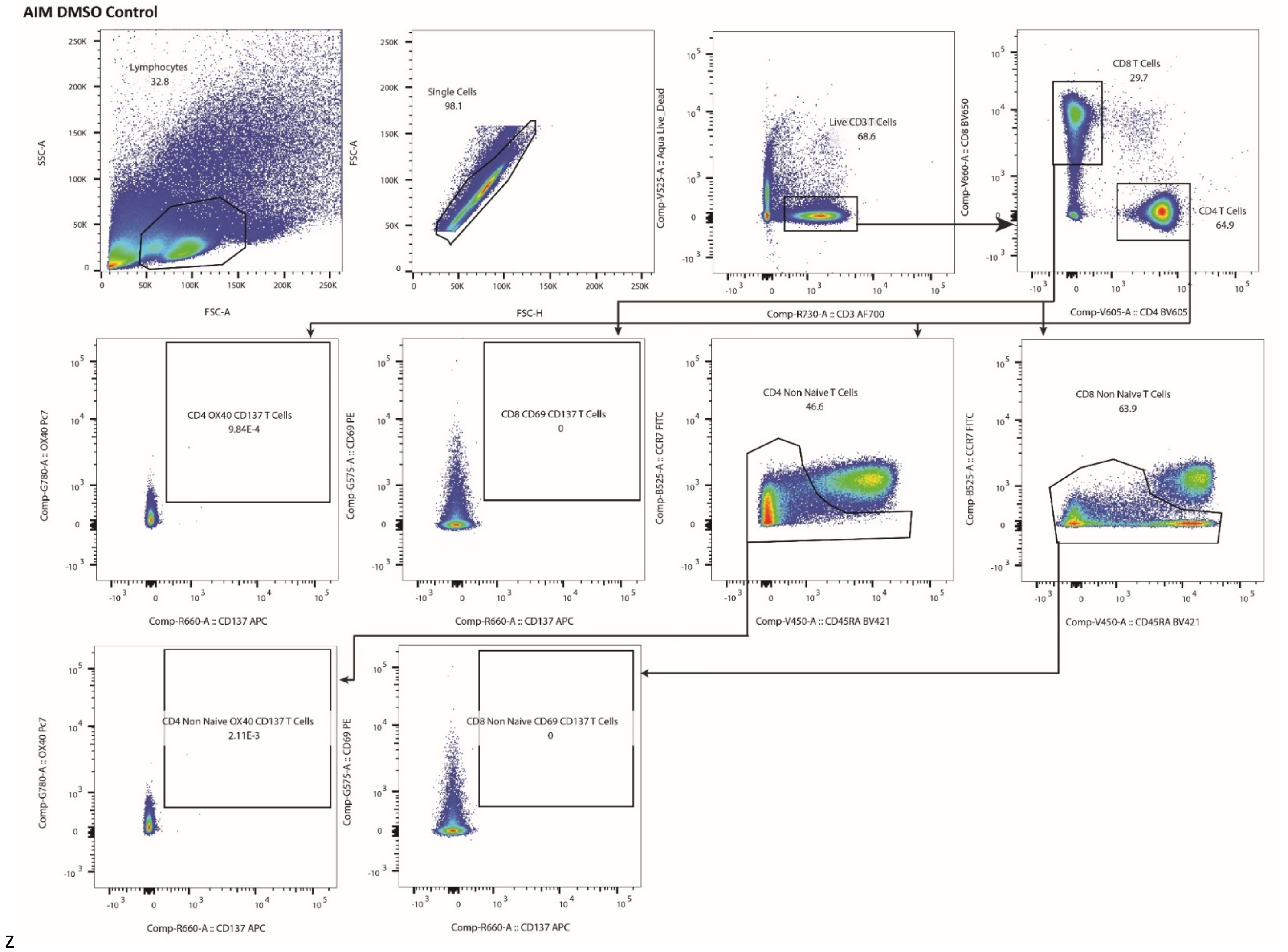

Supplementary Figure 1. Gating strategy for the Activation Induced Marker (AIM) assay. Data from memory (non naïve) CD4 and CD8 T cells were used in the primary analyses.

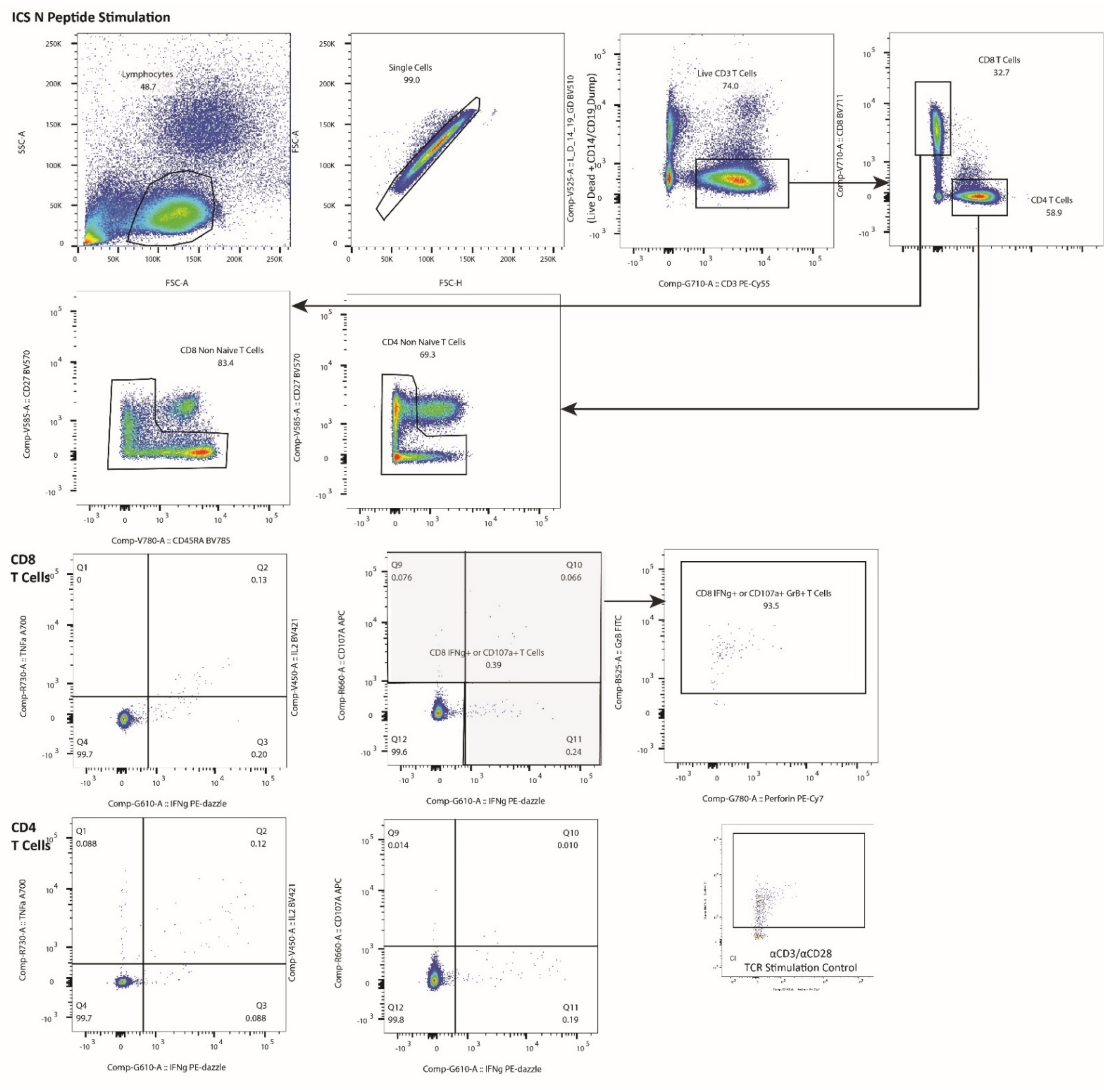

Supplementary Figure 2. Gating strategy for the Intracellular Cytokine Staining (ICS) assay. Data from memory (non naïve) CD4 and CD8 T cells were used in the analyses.

Supplementary Table 1. Participants with Activation Induced Marker (AIM) and Intracellular Cytokine Staining (ICS) Assay Results Greater than Pre-COVID-19 Control Samples Across All Timepoints.

Assay	N[a]	N Above Upper Interquartile Range (%+ Cells) of Pre-COVID-19 Controls (% of N)
CD4 AIM or ICS (N or S)	69	69 (100)
CD8 AIM or ICS (N or S)	69	66 (95.7)
CD4 AIM N or S	68	67 (98.5)
CD4 AIM N	68	67 (98.5)
CD4 AIM S	68	65 (95.6)
CD8 AIM N or S	68	52 (76.5)
CD8 AIM N	68	46 (67.6)
CD8 AIM 2	68	41 (60.3)
CD4 ICS N or S (IFNγ+/TNFα+ or IFNγ+)	65	65 (100)
CD4 ICS N	64	64 (100)
CD4 ICS S	64	63 (98.4)
CD8 ICS N or S (IFNγ+/TNFα+ or IFNγ+)	65	53 (81.5)
CD8 ICSN	65	47 (72.3)
CD8 ICS S	63	46 (73.0)

[a] Number of participants from the total analysis cohort (N=70) with valid AIM or ICS assay results (*e.g*. sufficient numbers of cells for analysis, adequate staining)

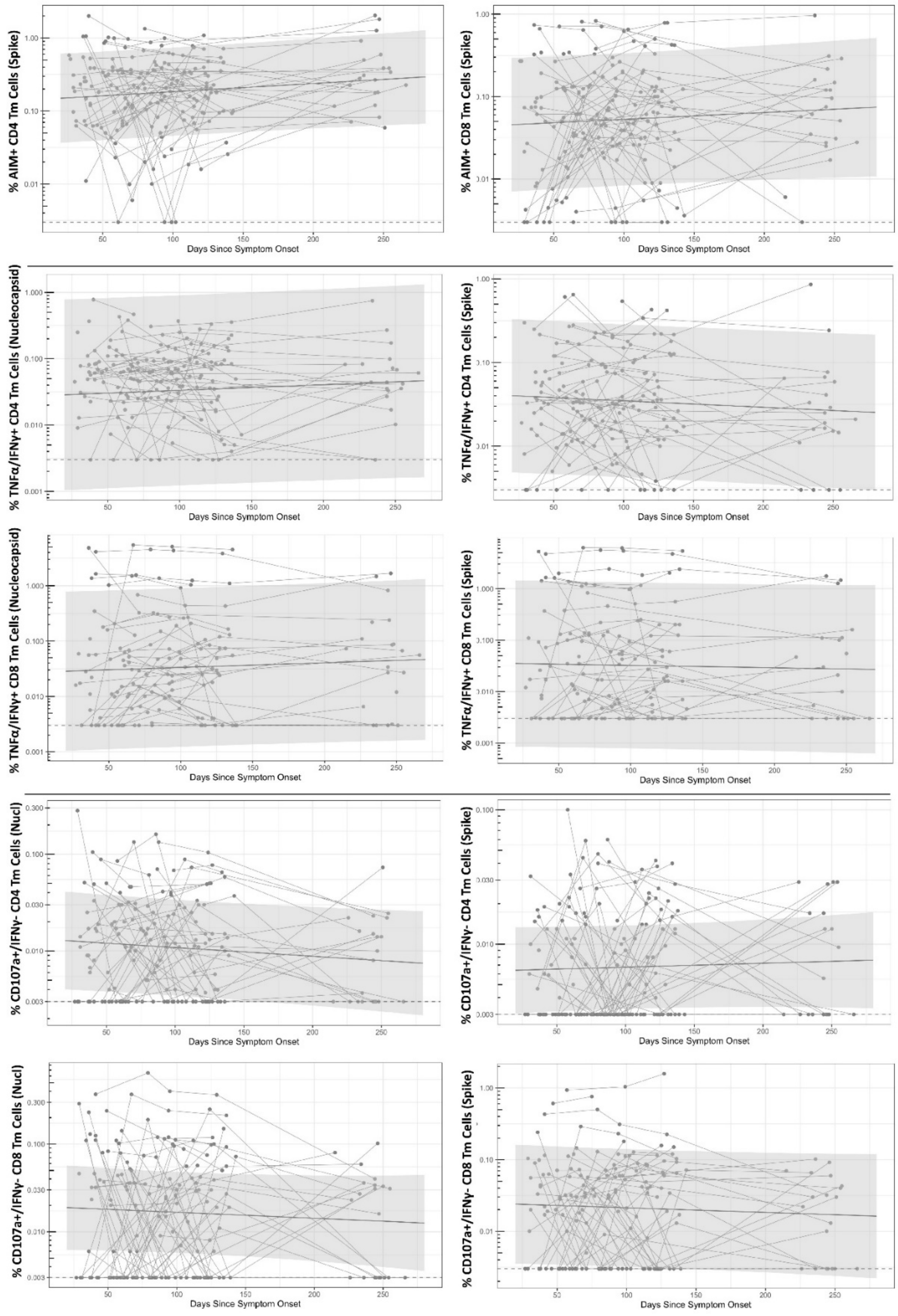
% AIM+ CD4 Tm Cells (Spike)
% AIM+ CD8 Tm Cells (Spike)
% TNFα/IFNγ+ CD4 Tm Cells (Nucleocapsid)
% TNFα/IFNγ+ CD4 Tm Cells (Spike)
% TNFα/IFNγ+ CD8 Tm Cells (Nucleocapsid)
% TNFα/IFNγ+ CD8 Tm Cells (Spike)
% CD107a+/IFNγ- CD4 Tm Cells (Nucl)
% CD107a+/IFNγ- CD4 Tm Cells (Spike)
% CD107a+/IFNγ- CD8 Tm Cells (Nucl)
% CD107a+/IFNγ- CD8 Tm Cells (Spike)
Days Since Symptom Onset

Supplementary Figure 3. Longitudinal T cell responses as measured by AIM SARS-CoV-2 Spike assay (**A**), dual expression of IFNγ+ and TNFα CD8+ and CD4+ T cells by ICS (**B**), and expression of CD107a (IFNγ negative) CD4+ and CD8+ T cells (**C**). Solid line and shaded region represent the median model prediction and 95% prediction interval from linear mixed effects modeling. Dashed lines represent assay limits of detection.

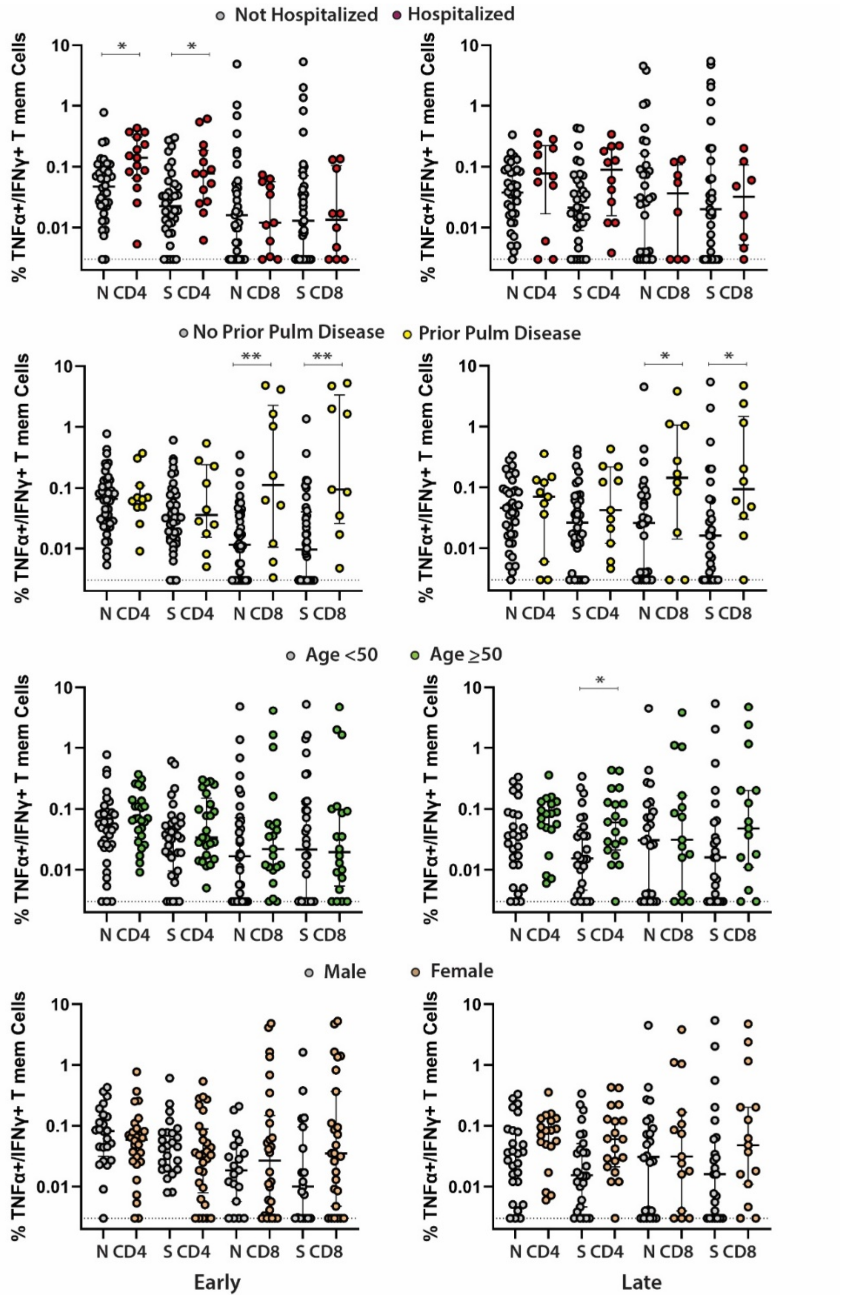

Supplementary Figure 4. Frequency of SARS-CoV-2-specific TNFα+/IFNγ+ T cell CD4+ T cells as measured by the ICS assay in study participants for various clinical and demographic factors at early (left panels, median 53 days after onset of symptoms) versus late (right panels, median 123 days from onset of symptoms) cross-sectional analysis time points. All data points are shown as individual points. Bars and lines in cross sectional data represent median values and interquartile ranges; (*) $P<0.05$, (**) $P<0.01$, (***) $P<0.001$ by non-parametric analyses.

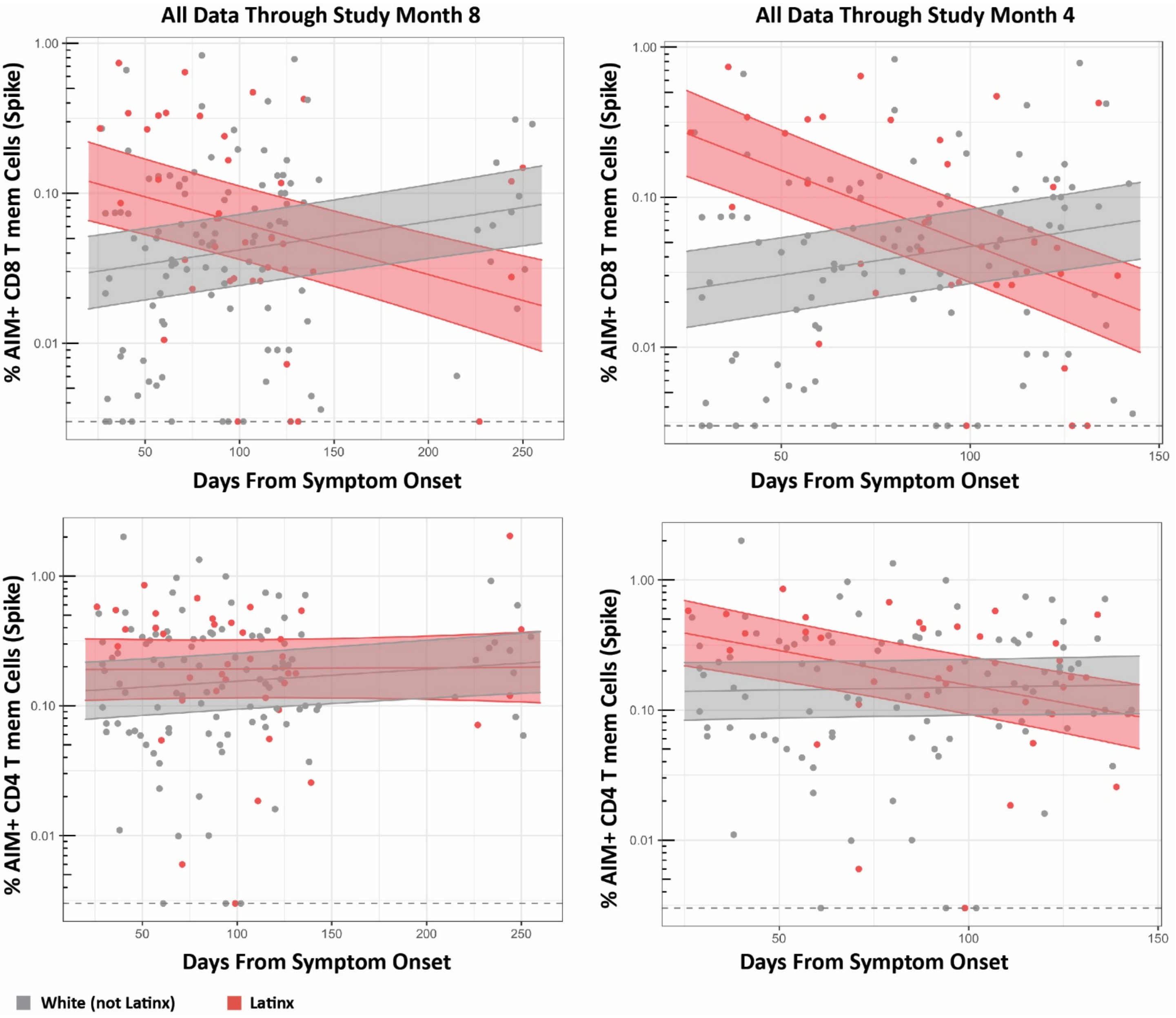

Supplementary Figure 5. Mixed effects models of longitudinal AIM+ CD8 T cell (top) and AIM + CD4 T cell (bottom) responses grouped by Latinx and white (non Latinx) ethnicity for all data through the month 8 collection time points are shown. In order to rule out potential batch effects, mixed effects models were similar including only data from month 4 time points (right panels). Solid line and shaded region represent the median model prediction and 50% prediction interval from linear mixed effects modeling. Dashed lines represent assay limits of detection.

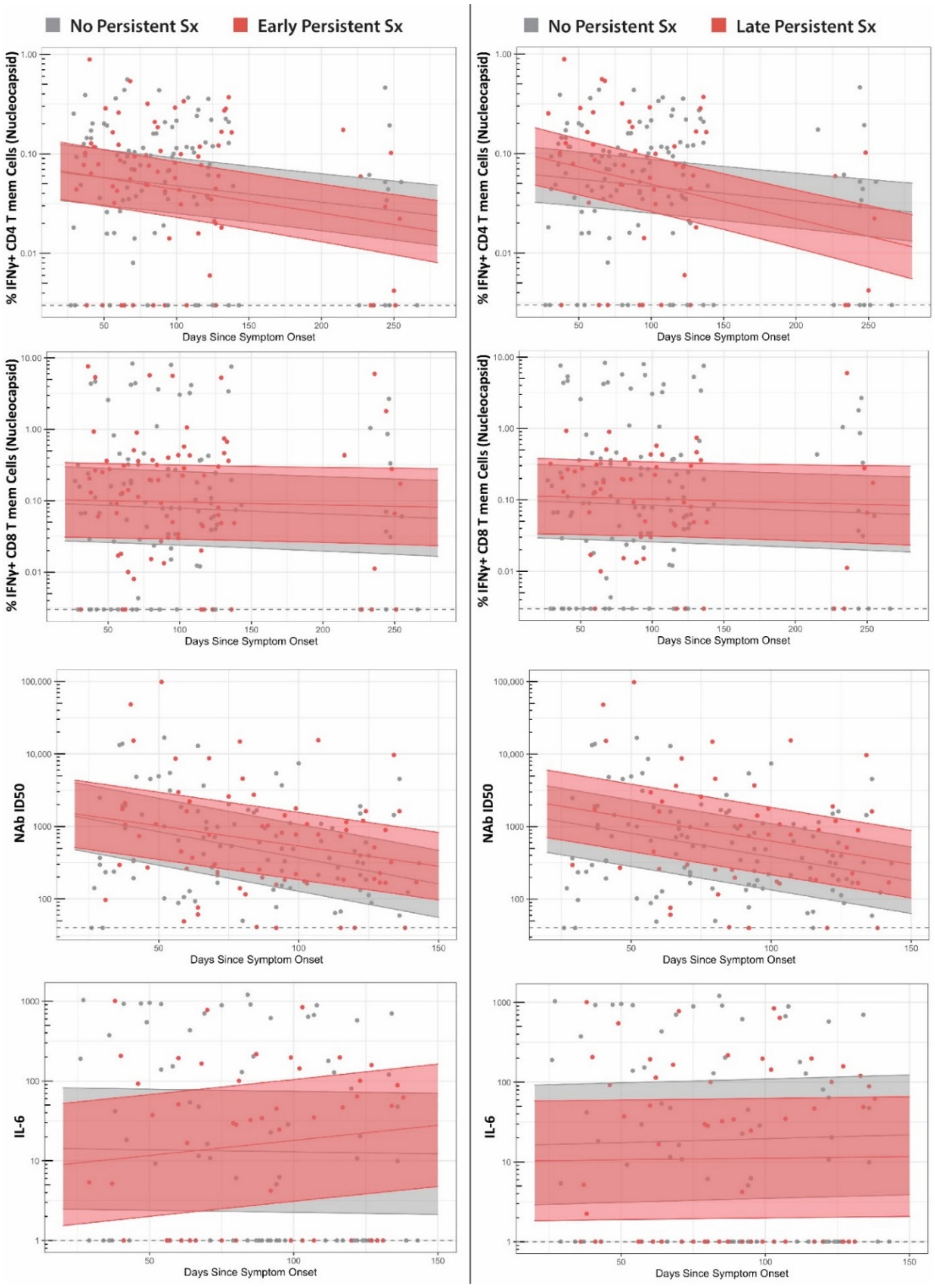

Supplementary Figure 6. Minimal differences in T cell, neutralizing antibody and inflammatory markers were observed between those with persistent following acute infection at the first study time point (left panels, median 53 days) and approximately 4 months after onset of acute illness (right panels, median 123 days). Solid line and shaded region represent the median model prediction and 50% prediction interval from linear mixed effects modeling. Dashed lines represent assay limits of detection. NAb = neutralizing antibody.

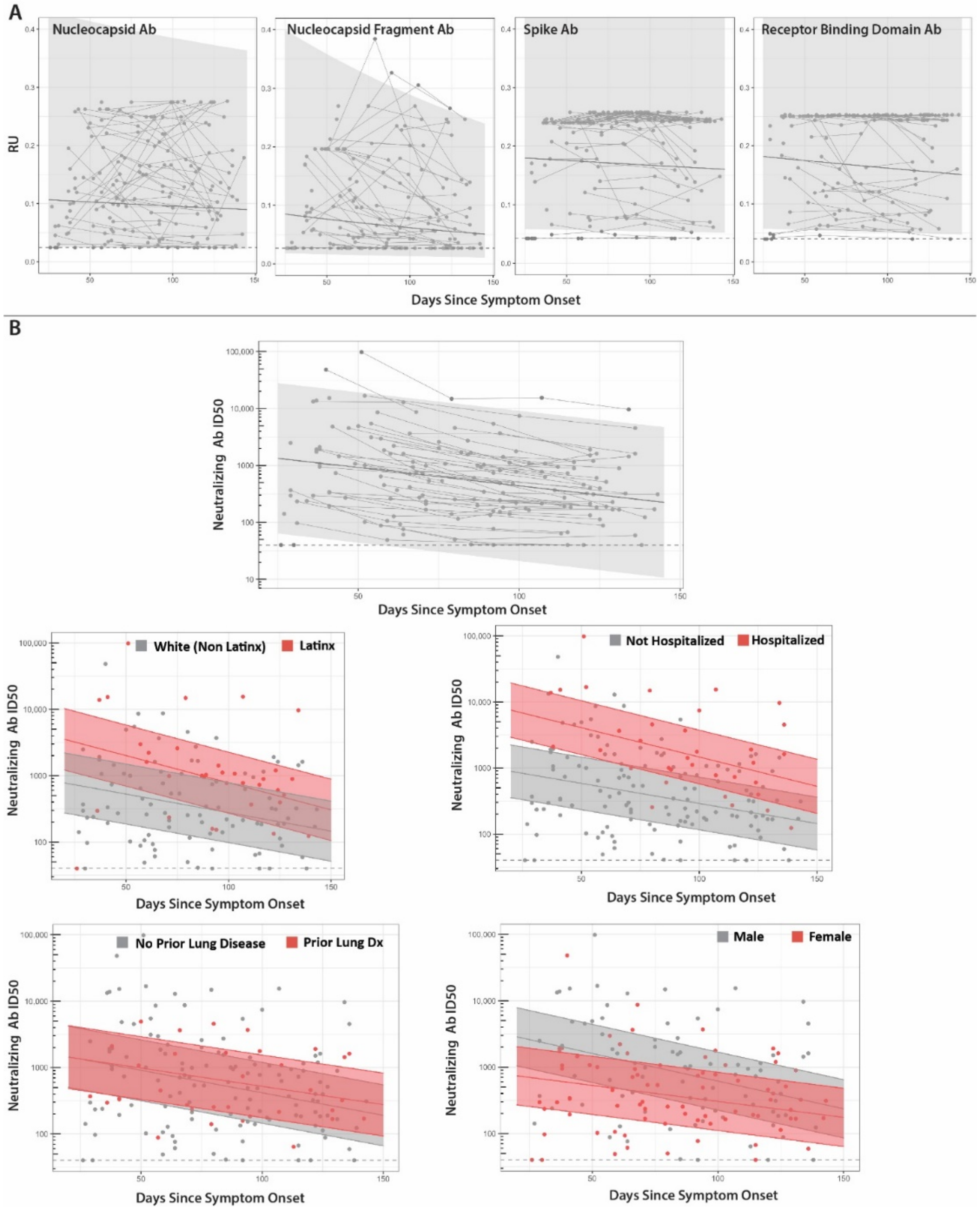

Supplementary Figure 7. Longitudinal antibody response (relative light units; RU) data are shown for data generating using the Luminex IgG assay across the nucleocapsid (N), nucleocapsid fragment (N.361), spike (S) and receptor binding domain (RBD) are shown in the top panels (**A**). Neutralizing capacity (**I**nfectious Dose, 50% [ID50] of Spike pseudovirus in presence of participant serum) for all participants and by various clinical and demographic factors are shown in the bottom panels (**B**). Points and connecting lines represent raw data for each individual. Solid line and shaded region represent the median model prediction and 95% prediction interval from linear mixed effects modeling (50% prediction intervals for comparisons between factors). Dashed lines represent assay limits of detection.

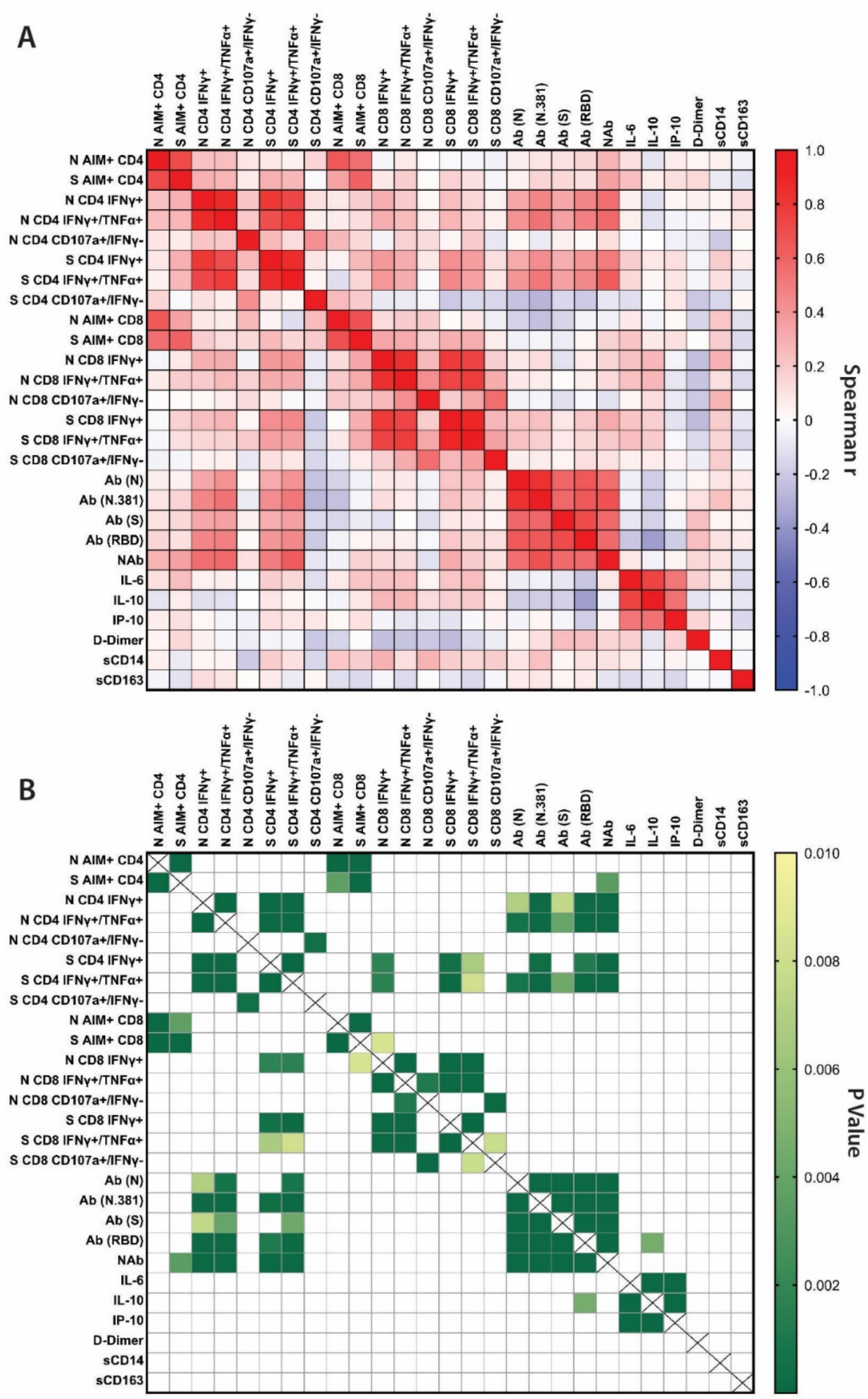

Supplementary Figure 8. Spearman correlation matrix of all T cell, antibody and inflammatory marker data is shown in (**A**). P value heatmap is shown in (**B**).

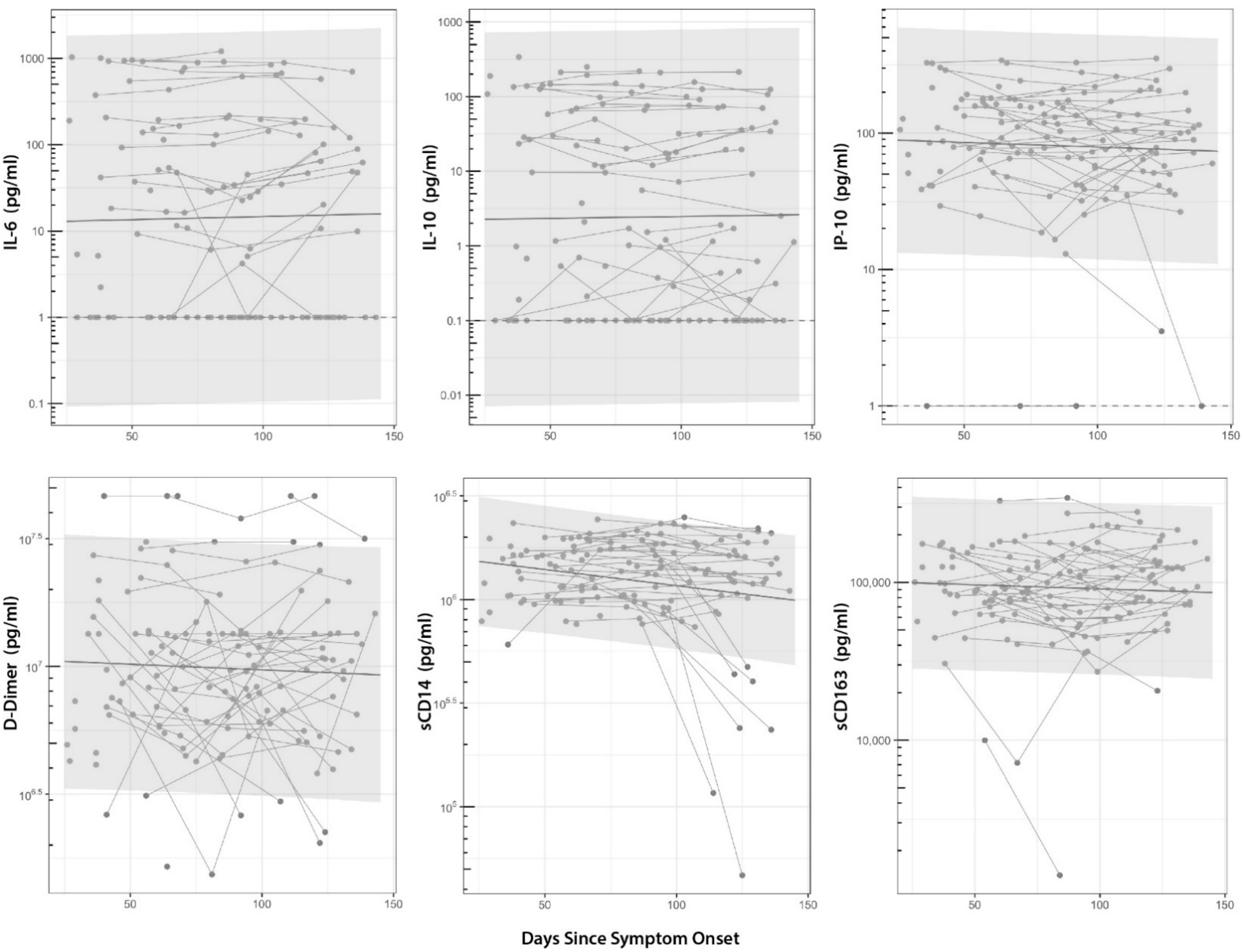

Supplementary Figure 9. Circulating markers of cytokines and markers of inflammation over time. With the exception of a modest decrease in sCD14 over time (1pg/ml per day (P=0.006), levels remained constant over 4 months following onset of COVID-19 symptoms. Solid line and shaded region represent the median model prediction and 95% prediction interval from linear mixed effects modeling

Supplementary Table 2. Antibodies Included in the Activation Induced Marker (AIM) Assay

Antibody		Fluorochrome	Clone/vendor/catalog
1	CD45RA	BV421	HI100/Biolegend/304130
2	CD14	V500	M5E2/BD/561391
3	CD19	V500	HIB19/BD/561121
4	Live/Dead	Aqua	Invitrogen/L34966
5	CD8	BV650	RPA-T8/BioLegend/301042
6	CD4	BV605	RPA-T4/BD/562659
8	CCR7	FITC	G043H7/Biolegend/353216
9	CD69	PE	FN50/BD/555531
10	OX40	PE-Cy7	Ber-ACT35/Biolegend/350012
11	CD137	APC	4B4-1/BioLegend/309810
12	CD3	AF700	OKT3/Biolegend/317340
13	CD40L	PE-Dazzle	24-31/Biolegend/310840

Supplementary Table 3. Antibodies Included in the Intracellular Cytokine Staining (ICS) Assay

Antibody		Fluorochrome	Clone/vendor/catalog
1	IL-2	BV421	MQ1-17H12/Biolegend/500328
2	CD14	BV510	M5E2/Biolegend/301842
3	CD19	BV510	HIB19/Biolegend/302242
4	TCR g/d	BV510	B1/Biolegend/331220
5	Live/Dead	Aqua	Invitrogen/L34966
6	CD27	BV570	O323/BioLegend/302825
7	CD4	BV650	OKT4/Biolegend/317436
8	CD8	BV711	SK1/Biolegend/344733
9	CD45RA	BV785	HI100/Biolegend/304140
10	GranB	FITC	Ber-ACT35/Biolegend/350012
11	IFNg	PE-CF594	GB11/Biolegend/515403
12	CD3	PE-Cy5.5	SK7/eBioscienc/35-0036-42
13	CD107a	APC	H4A3/Biolegend/328620
14	TNFa	AF700	6402/R&Dsystems/IC9677N-100UG
